# Funding source and the quality of reports of chronic wounds trials: 2004 to 2011

**DOI:** 10.1186/1745-6215-15-19

**Published:** 2014-01-14

**Authors:** Robert Hodgson, Richard Allen, Ellen Broderick, J Martin Bland, Jo C Dumville, Rebecca Ashby, Sally Bell-Syer, Ruth Foxlee, Jill Hall, Karen Lamb, Mary Madden, Susan O’Meara, Nikki Stubbs, Nicky Cullum

**Affiliations:** 1 Department of Health Sciences, University of York, Seebohm Rowntree Building, YO10 5DD Heslington, England; 2 School of Nursing, Midwifery and Social Work, University of Manchester, Jean McFarlane Building, Oxford Road, M13 9PL Manchester, England; 3 NHS Leeds Community Health Care, St Mary’s Hospital, Green Hill Road, Leeds, UK, England; 4 School of Healthcare, Baines Wing, University of Leeds, Leeds, UK, England

**Keywords:** Wound care, Randomised controlled trials, Bias, Industry funding

## Abstract

**Background:**

Critical commentaries suggest that wound care randomised controlled trials (RCTs) are often poorly reported with many methodological flaws. Furthermore, interventions in chronic wounds, rather than being drugs, are often medical devices for which there are no requirements for RCTs to bring products to market. RCTs in wounds trials therefore potentially represent a form of marketing. This study presents a methodological overview of chronic wound trials published between 2004 and 2011 and investigates the influence of industry funding on methodological quality.

**Methods:**

A systematic search for RCTs for the treatment of chronic wounds published in the English language between 2004 and 2011 (inclusive) in the Cochrane Wounds Group Specialised Register of Trials was carried out.

Data were extracted on aspects of trial design, conduct and quality including sample size, duration of follow-up, specification of a primary outcome, use of surrogate outcomes, and risks of bias. In addition, the prevalence of industry funding was assessed and its influence on the above aspects of trial design, conduct and quality was assessed.

**Results:**

A total of 167 RCTs met our inclusion criteria. We found chronic wound trials often have short durations of follow-up (median 12 weeks), small sample sizes (median 63), fail to define a primary outcome in 41% of cases, and those that do define a primary outcome, use surrogate measures of healing in 40% of cases. Only 40% of trials used appropriate methods of randomisation, 25% concealed allocation and 34% blinded outcome assessors. Of the included trials, 41% were wholly or partially funded by industry, 33% declared non-commercial funding and 26% did not report a funding source. Industry funding was not statistically significantly associated with any measure of methodological quality, though this analysis was probably underpowered.

**Conclusions:**

This overview confirms concerns raised about the methodological quality of RCTs in wound care and illustrates that greater efforts must be made to follow international standards for conducting and reporting RCTs. There is currently minimal evidence of an influence of industry funding on methodological quality although analyses had limited power and funding source was not reported for a quarter of studies.

## Background

Chronic wounds, of which venous leg ulcers, pressure ulcers and foot ulcers make up the majority [1], are prevalent conditions that have considerable impact on patients’ quality of life and are costly to treat. For example, venous leg ulcers are estimated to affect 15 to 18 in 1,000 people in the United Kingdom (UK) and cost between £300 and £600 million (UK pounds) per year in health expenditure [2]. Despite the clinical and financial significance of chronic wounds, a number of commentators have expressed concerns about both the quantity and quality of RCTs within the broader area of wounds research [3-5].

RCTs within the field of chronic wounds, as within all areas of medicine, represent an essential part of this evidence base due to their ability to provide unbiased estimates of relative treatment effects. However, the generation of such unbiased estimates depends on adequate methodological measures being taken to minimise bias. For example, it has been demonstrated that failure to conceal allocation and failure to blind outcome assessors and participants results in biased, larger estimated effect sizes [6-12]. The failure to specify a primary outcome *a priori* can also introduce bias as authors are free to cherry pick outcomes for reporting that show a statistically significant difference [13]. As well as methodological features of RCTs that can minimise bias, other elements of study design also impact the overall quality of evidence generated. These design features include sample size, choice of primary outcome and the duration of post-randomisation follow-up. Trial sample size is important because while estimates from small trials are not necessarily biased, they will be under-powered to detect anything but the largest treatment effects, which also leads to small trials being associated with publication bias [14]. The choice of primary outcome is similarly important and needs to be meaningful to both clinicians and patients. Duration of follow-up is linked to issues of study power and outcome selection. Important outcomes such as wound healing can take a long time to achieve (often months for chronic wounds). Studies with a short duration of follow-up will potentially miss outcome events and be underpowered. One approach is the selection of other surrogate outcomes that can be measured over a shorter period but which may not be valid proxies for, or may be harder to interpret than, clinically meaningful outcomes.

The importance of methodological characteristics, such as those outlined above, has led to a number of studies being conducted that sought to quantify the methodological quality on both samples of RCTs from across medicine [15,16] and within specific areas [17-20]. However, despite the methodological concerns described above, no such overview exists within the area of chronic wounds - an area of medicine where most treatments are medical devices for which RCT data are not necessary for licensing and marketing.

Previous research in other areas of medicine suggests that funding can have an important impact on a number of trial characteristics. For example, studies have observed that industry-funded drug trials are more likely to report in favour of (the sponsored) treatment when compared with research not funded by a commercial organisation [21-24]. Studies have also shown commercial funding to be associated with shorter duration of follow-up, [25] more frequent use of non-active comparators [26,27] and a tendency to be at a lower risk of bias [24,28-30]. In the area of chronic wounds, there is little data regarding the prevalence of industry-funded trials or the influence of industry funding on trial design. This study is a methodological review of recent trial evidence in the field of chronic wounds. It aims to assess the prevalence of industry-funded trials in chronic wound care and identify whether funding source is associated with features of methodological quality.

## Methods

This study was protocol-driven with all search terms, selection criteria and analysis pre-specified.

### Selection

A library of eligible RCTs in chronic wound care was assembled and used. Eligible studies were:

1. Randomised comparisons of treatments for foot, leg or pressure ulcers in any setting and for any category of patient (studies that included other wound types as well were excluded)

2. Published between 2004 and 2011 (inclusive)

3. Reported in English (due to a lack of translation resources)

Studies were excluded if they were:

1. Secondary reports, where the primary report of the main study was referenced

2. Conference abstracts (since these typically do not contain sufficient methodological information in order to assess risk of bias (RoB)

3. A protocol of a trial

4. A cost effectiveness study (unless they also reported the effectiveness of an intervention)

5. A phase 1 trial (since these are not aimed at determining effectiveness)

Trials were considered to be a RCT and included if they were described as ‘randomised’ either in the title or in the text of the paper. If no randomisation process was referred to, the study was excluded.

This library was constructed by searching the Cochrane Wounds Group Specialised Register of Trials. The Register is maintained by the Cochrane Wounds Group and aims to identify all randomised and quasi-randomised controlled trials in the area of wounds research. Reports are identified for inclusion in the register by regular searches of a number of databases including Medline, Embase, CINAHL and Central along with periodic searches of other databases. Studies included in the register have been coded on several criteria including wound type. A search was therefore carried out using the following search terms in the condition field: Pressure* or Venous or Leg* or Ulcer* or Diabet*, and intervention field: Treat*.

The titles and abstracts (where available) of the identified studies were screened by a single author to exclude studies which obviously did not meet the inclusion criteria. The full text was obtained for the all studies that were potentially relevant. Two authors independently checked the full papers for eligibility. Any disagreements were resolved through discussion, and where agreement could not be reached, a third author acted as arbitrator.

### Data extraction

For each eligible study relevant data were initially extracted by one of seven authors. A second independent extraction was completed by a single author (blinded to the initial extraction). Any disagreements were resolved through discussion. Where agreement could not be reached, a third author arbitrated. The process of data extraction was extensively piloted with data recorded on a Microsoft Access database (Microsoft, Redmond, WA, USA - http://office.microsoft.com/en-gb/access/), using drop-down menus where possible.

Data extracted included the publishing journal, impact factor of publishing journal, journal specialty, wound type, study design (parallel or other), number of randomised groups, sample size, duration of follow-up, funding source, primary outcome if defined, and information on the interventions and comparators investigated (further information on decision rules for data classification can be found in Additional file 1). Risk of bias was assessed using the Cochrane risk of bias tool [31,32] for the domains sequence generation, allocation concealment and blinding of outcome assessors. An overall assessment risk of bias was also made following Cochrane guidelines [32]. See Table 1 for definitions used in assessments.

**Table 1:** Definitions used in assessment

**Primary outcome**	Primary or main outcomes defined explicitly, or an outcome used in power calculation, or a main outcome described explicitly in primary study objectives
**Type of outcome**	Primary outcomes where defined were classified as complete healing if primary outcome was proportion healed or time to complete healing; surrogate healing if the primary outcome was any other healing-related outcome; and, non-healing if the primary outcome was a non-healing outcome such as presence of infection or pain.
**Sequence generation**	Method described for generating the randomisation sequence used to allocate participants to study groups, including computer-generated sequences, random number tables, and coin tosses
**Allocation concealment**	Method described to prevent the individual responsible for enrolling trial participants from knowing or predicting the allocation sequence in advance, including central randomisation or sealed, opaque, sequentially numbered envelopes
**Blinding of assessors**	Outcome assessors had no knowledge of the participants’ group allocation or it was judged that the outcome and the outcome measurement was unlikely to be influenced by lack of blinding (for example, mortality).
**Overall risk**	- Low risk of bias: Study was at low risk of bias in all three domains.
	**-** Unclear risk of bias: Study at unclear risk of bias in one or more domain and at high risk of bias in none.
	**-** High risk of bias: Study was at high risk of bias in one or more domains.

### Data analysis

Descriptive summary statistics were calculated for each of the general and methodological items specified, and outcomes were stratified by wound type and by funding source. The descriptive summary statistics were then used to compare the methodological quality of commercially funded and non-commercially funded randomised trials for which mean differences or odds ratios (OR) (as appropriate) with 95% confidence intervals (CI) were calculated. To assess difference in study duration between studies with different funding sources, mean differences were logged. Results were initially recorded in Microsoft Access (Microsoft, Redmond, WA, USA - http://office.microsoft.com/en-gb/access/) and Stata 12 (StataCorp, College Station, TX, USA – http://www.stata.com) was used for data analysis.

## Results

**Figure 1:**
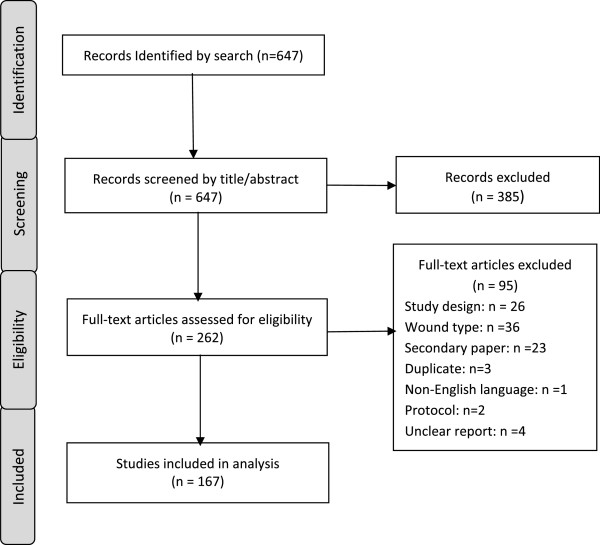
Overview of review process.

The results of the study selection process and reasons for exclusion are presented in Figure 1. We identified 647 potentially eligible studies. After an initial assessment of titles and abstracts, 385 studies were excluded and full text copies of the remaining 262 potentially eligible studies were obtained. Ninety-five were excluded for the following reasons: 36 concerned an ineligible wound type; 26 had an ineligible study type/design; three were duplicate publications of existing studies; 23 were secondary reports of included studies; 1 paper was not in English; 2 were trial protocols; and 4 studies were excluded due to exceptionally poor reporting, which made judgement of whether the study met the inclusion criteria impossible. The remaining 167 studies met the inclusion criteria.

Table 2 presents the general characteristics of the 167 included studies stratified by wound type. Leg ulcers were the most frequently investigated of the three wound types (38%; 63/167), closely followed by foot ulcers (34%; 57/167). Only 19% (31/167) of trials focused on pressure ulcers over the eight-year period investigated. A further 9.6% (16/167) of trials investigated more than one wound type.

**Table 2:** General characteristics of included studies

	**All (n = 167)**	**Leg ulcers (n = 63)**	**Pressure ulcers (n = 31)**	**Foot ulcers (n = 57)**
**Journal type**				
General medical	11 (6.6%)	3 (4.8%)	4 (13%)	2 (3.5%)
Wounds specialty	57 (34%)	26 (41%)	10 (32%)	13 (23%)
Non-wounds specialty	99 (59%)	34 (54%)	17 (55%)	42 (74%)
**Impact factor**				
Median (IQ range)	1.93 (1.21 to 3.01)	2.38 (1.26 to 3.21)	1.63 (1.36 to 2.48)	1.48 (0.799 to 2.90)
**Funding**				
Commercial	58 (35%)	24 (38%)	7 (23%)	16 (28%)
Mixed	10 (6.0%)	3 (4.8%)	2 (6.5%)	5 (8.8%)
Non-commercial	55 (33%)	20 (32%)	14 (45%)	16 (28%)
Unclear/not reported	44 (26%)	16 (25%)	8 (26%)	20 (35%)
**Study design**				
Other				
Parallel	160 (96%)	58 (92%)	30 (97%)	56 (98%)
	7 (4.2%)	5 (7.9%)	1 (3.2%)	1 (1.8%)
**Study groups**^**a**^				
2	144 (90%)	52 (90%)	27(90%)	51 (91%)
3	14 (8.8%)	6 (10%)	2 (6.7%)	4 (7.0%)
4+	2 (1.3%)	0 (0.0%)	1 (3.3%)	1 (1.8%)
**Intervention**				
Bandages/stockings	14 (8.4%)	14 (22%)	0 (0.0%)	0 (0.0%)
Dressings/topical Agents	42 (25%)	20 (32%)	10 (32%)	10 (18%)
Drugs	33 (20%)	8 (13%)	4 (13%)	16 (28%)
Growth factors	16 (9.6%)	0 (0.0%)	0 (0.0%)	13 (23%)
Tissue grafts	11 (6.6%)	4 (6.3%)	1 (3.2%)	5 (8.8%)
Other	51 (31%)	17 (27%)	16(52%)	13(23%)
**Comparators**				
Placebo	50 (30%)	14 (22%)	12 (39%)	18 (32%)
Usual/standard Care	43 (26%)	20 (32%)	8 (26%)	12 (21%)
Named comparison	74 (44%)	29 (46%)	11 (35%)	27 (47%)

^a^Parallel trials only.

The majority of trials were parallel in design (96%; 160/167), with only 4.2% (7/167) using other trial designs (either crossover or factorial). Most (parallel) trials had just two intervention arms (90%; 144/160); 8.8% (14/160) had three arms, and 1.3% (2/160) had four arms. The most frequent intervention types investigated were dressings and topical agents (25%; 42/167), drugs (20%; 33/167), growth factors (9.6%; 16/167), bandages and stockings (8.4%; 14/167), and tissue grafts (6.6%; 11/167). There were a further 23 different technologies investigated.

The majority of trials were reported in specialty journals (93%; 156/167), with 34% (57/167) published in wounds journals. The remaining studies (7.0%, 11/167) were published in general medical journals. Of the included studies, 77% (128/167) were published in a journal with an ISI impact factor. The median impact factor for the 128 studies was 1.93, (interquartile range (IQR 1.21 to 3.01)).

In total, 35% (58/167) of studies were reported as having been commercially funded, 33% (55/167) were not commercially funded, and 6.0% (10/167) had funding from both commercial and noncommercial sources. The remaining 26% (44/167) of studies either did not report the source of funding, or the status of the funding source was unclear.

Table 3 presents information on a number of methodological characteristics of the included trials. The median number of participants was 60 (IQR 35 to 99), with a median of 28 (IQR 16 to 48) participants per treatment arm.

**Table 3:** Methodological characteristics of included studies

	**All (n = 167)**	**Leg ulcers (n = 63)**	**Pressure ulcers (n = 31)**	**Foot ulcers (n = 57)**
**Number of participants(median (IQ range))**				
Overall	60 (35 to 99)	81 (43 to 126)	44 (26 to 60)	50 (30 to 86)
Per treatment arm	28 (16 to 47)	37 (21 to 62)	21 (7 to 30)	27 (15 to 43)
**Duration(in months; median (IQ range))**				
All	2.8 (1.6 to 5.6)	3.0 (2.8 to 6.0)	1.9 (0.9 to 3.3)	3.0 (1.4 to 4.7)
PO: Complete healing	5.6 (3.0 to 6.1)	6 (3.0 to 12)	3.9 (1.3 to 6)	4.7 (4.7 to 5.6)
PO: Surrogate healing	2.8 (1.7 to 3.7)	2.8 (1.9 to 3.0)	1.6 (0.93 to 2.8)	1.9 (1.1 to 3.4)
PO: Nonhealing	1.7 (1.4 to 2.1)	1.9 (1.6 to 36)	1.4 (1.4 to 1.4)	1.9 (1.4 to 3.9)
PO: Not defined	2.8 (1.3 to 4.3)	3 (1.6 to 5.6)	1.8 (0.85 to 3.0)	2.8 (0.93 to 3.7)
**Primary outcome**				
Complete Healing	40 (24%)	18 (29%)	4 (13%)	16 (28%)
Surrogate Healing	47 (28%)	15 (24%)	14 (45%)	12 (21%)
Non-healing	11 (6.6%)	3 (4.8%)	1 (3.2%)	6 (11%)
None	69 (41%)	27 (43%)	12 (39%)	23 (40%)
**Sequence generation**				
Low RoB	67 (40%)	27 (43%)	15 (48%)	19 (33%)
Unclear RoB	98 (59%)	36 (57%	15 (48%)	37 (65%)
High RoB	2 (1.2%)	0 (0.0)%	1 (3.2%)	1 (1.6%)
**Allocation concealment**				
Low RoB	41 (25%)	22 (35%)	5 (16%)	11 (19%)
Unclear RoB	123 (74%)	41 (65%)	24 (77%)	45 (80%)
High RoB	3 (1.8%)	0 (0.0%)	2 (6.5%)	1 (1.6%)
**Blinding of assessors**				
Low RoB	56 (34%)	13 (21%)	15 (48%)	23 (40%)
Unclear RoB	64 (38%)	24 (38%)	13 (42%)	22 (39%)
High RoB	47 (28%)	26 (41%)	3 (9.7%)	12 (21%)
**Overall RoB**				
Low RoB	10 (6.0%)	4 (6.3%)	2 (6.5%)	3 (5.3%)
Unclear RoB	107 (64%)	33 (52%)	24 (77%)	41 (72%)
High RoB	50 (30%)	26 (41%)	5 (16%)	13 (23%)

PO, primary outcome; RoB, risk of bias.

The median duration of follow-up of the trials was 12 weeks (IQR 6.7 to 24) and varied significantly depending on the nature of the primary outcome. Studies reporting surrogate measures of healing (for example, change in ulcer size) had a median follow-up of 12 weeks (IQR 7.3 to 15.9) compared with 24 weeks (IQR 12.9 to 26.1) for studies with a primary outcome of either time to complete healing or proportion of wounds completely healed.

The proportion of trials that defined a primary outcome measure was 59% (98/167). Of those that defined a primary outcome, 41% (40/98) reported a measure of complete healing as the primary outcome (either time to complete healing or proportion of wounds completely healed), 48% (47/98) reported an intermediate (surrogate) measure of healing (for example, change in ulcer size/area) and 11% (11/98) of trials reported a primary outcome that was unrelated to healing (for example, pain).

The Cochrane risk of bias assessment [30,31] revealed that of the 167 included trials, only 40% (67/167) described an appropriate method of random sequence generation; 59% (98/167) were unclear or did not report the method of sequence generation and 1.2% (2/167) reported using inappropriate methods of sequence generation such as alternation or date of birth. Only 25% (41/167) of trials reported adequate methods of allocation concealment, such as remote telephone randomisation or sealed, opaque, sequentially numbered envelopes; 74% of (123/167) trials were unclear or did not report their method of allocation concealment; and 1.8% (3/167) did not adequately conceal allocation. With regards to blinding of outcome assessors, 34% (56/167) of trials were classified as at low risk of bias, 38% (64/167) were at unclear risk and 28% (47/167) of trials were at high risk of bias. Following Cochrane review guidelines [31] to construct an overall risk of bias assessment, 30% (50/167) of trials were judged to be at high risk of bias, 64% (107/167) at unclear risk of bias and just 6.0% (10/167) at low risk of bias.

Table 4 presents the methodological characteristics of the included studies stratified by funding source. The sample sizes of commercially funded trials were not statistically significantly different from those of non-commercially funded trials (difference 8.0 participants, 95% CI −25 to 41). Differences in duration were modelled using a log model due to the data being highly skewed. There was no statistically significant difference in the duration of follow-up between commercially funded and non-commercially funded trials (with commercially funded studies having, on average −1.3 months, less follow-up , 95% CI −1.8 to 1.06 months). Commercially funded trials were not more or less likely to specify a primary outcome than non-commercially funded trials (OR 1.48, 95% CI .74 to 2.94), and were no more likely to identify a surrogate healing measure as their primary outcome (OR 0.68, 95% CI 0.29 to 1.57). There was no statistically significant difference between commercially funded and non-commercially funded trials in the likelihood of being classified as at either high or unclear risk of bias in the domains of sequence generation (OR 0.63, 0.34 to 1.16), allocation concealment trials (OR 1.08, 95% CI 0.62 to 1.88) or blinding of outcome assessors (OR 0.84, 95% CI 0.47 to 1.50). There was no statistically significant difference between industry-funded and nonindustry-funded trials in the odds of being classified as at high or unclear risk of bias overall (OR 0.91, 95% CI 0.54 to 1.52).

**Table 4:** Methodological characteristics of included studies by funding type

	**Fully or partly commercially funded**	**Non-commercially funded**	**Funding unclear/not reported**
	**(n = 68)**	**(n = 55)**	**(n = 44)**
**Number of participants(median (IQ range))**			
Overall	63 (40 to 117)	52 (28 to 91)	60 (29 to 98)
Per treatment arm	30 (18 to 51)	26 (14 to 44)	23 (14 to 41)
**Duration (in months; median (IQ range))**			
Overall	2.8 (1.9 to 5.6)	3.0 (1.9 to 6.0)	2.0 (1.2 to 3.5)
**Primary outcome**			
Complete healing	17 (25%)	15 (27%)	8 (18%)
Surrogate healing	26 (38%)	11 (20%)	10 (23%)
Non-healing	5 (7.4)	4 (7.3%)	2 (4.5%)
None	20 (29%)	25 (45%)	24 (55%)
**Sequence generation**			
Low RoB	23 (34%)	31 (56%)	14 (32%)
Unclear RoB	43 (63%)	24 (44%)	30 (68%)
High RoB	2 (2.9%)	0 (0.0)%	0 (0.0%)
**Allocation concealment**			
Low RoB	21 (31%)	13 (24%)	7 (16%)
Unclear RoB	45 (66%)	41 (75%)	37 (84%)
High RoB	2 (2.9%)	1 (1.8%)	0 (0.0%)
**Blinding of assessors**			
Low RoB	23 (34%)	23 (42%)	10 (23%)
Unclear RoB	19 (28%)	20 (36%)	25 (57%)
High RoB	26 (38%)	12 (22%)	9 (20%)
**Overall RoB**			
Low RoB	3 (4.4%)	5 (9.1%)	2 (4.5%)
Unclear RoB	37 (54%)	37 (67%)	33 (75%)
High RoB	28 (41%)	13 (24%)	9 (20%)

RoB, risk of bias.

## Discussion

### Key findings

This study aimed to describe a number of methodological characteristics of trials in chronic wounds published during the period from 2004 to 2011 and identify whether funding source was associated with the methodological quality of trials. Trial reports in chronic wounds tend to be published in comparatively low impact journals with a significant proportion of studies being published in journals with no published impact factor.

The average sample size observed in our sample of trials was very small. The statistical power of a hypothetical trial involving 60 participants (the median sample size), where approximately 50% of ulcers heal in 12 weeks [32] (the median duration of follow-up) and where it is assumed there is a modest effect size of 15% [32], would be just 21%. In other words, only one in five of the trials in our sample are sufficiently large to detect a statistically significant treatment effect should one exist. The vast majority of chronic wounds trials are therefore underpowered to detect all but very large effects. While there is some debate regarding the importance of adequately powering a trial to detect a difference, [33-35], there is evidence that published small trials yield larger effect sizes than large trials [14], most likely as a result of publication bias. The fact that many chronic wounds trials are small means that it is very important that systematic reviewers check for publication bias wherever possible. Equally, it is important that authors register trials so that reviewers can identify on-going and potentially unpublished material.

Whilst many trials specified a primary outcome, a significant proportion did not. There were opportunities for authors to cherry pick results (that is, presenting outcomes for which their statistically significant differences). In a related study, Lockyer *et al.*[36] found that 86% of wound trials that did not define a primary outcome claimed a significant treatment effect. This raises deep suspicion of bias given the inadequate statistical power of trials in this field.

Our results show that the use of surrogate healing measures (for example, change in ulcer size) is widespread and indeed the majority of studies that define a primary outcome use these intermediate measures of healing. The cost of longer term follow-up is likely to be the primary reason for this. Clearly such cost reductions represent an advantage of utilising surrogate outcomes, and there is good evidence that a number of surrogate endpoints such as initial change in ulcer size are good predictors for the clinically meaningful endpoint of complete healing [37-39]. There are, however, a number of reasons to remain cautious about using surrogate endpoints. Firstly, surrogate measure of healing such as change in ulcer size or area introduce the complex issue of how to measure ulcer size and opens the potential for inaccuracies and lack of reliability in systems of measurement. A recent systematic review into the performance of instruments for the measurement of pressure ulcers revealed little evidence to support the reliability of different methods of measurement, particularly with regard to ulcer depth and volume [40]. Secondly, while some studies have shown surrogate healing measures to be good predictors of complete healing, what is less clear is their ability to differentiate between treatment effects. It is not enough that a surrogate predicts the clinical endpoint, it must also be able to predict changes in the clinical endpoint due to different treatment effects [41,42].

The results of the risk of bias assessments raise a number of concerns. Across all three bias domains, a minority of trials were at low risk of bias. Assessment of bias was, however, hindered by poor reporting with the vast majority of studies at unclear risk for at least one domain. While it is encouraging that only a small minority of studies were at high risk of bias for either sequence generation or allocation concealment, it is impossible to make any judgement about the prevalence of high risk of bias trials in these domains when such a large proportion of trials are poorly reported. Previous research has suggested that reporting is worse than conduct [43,44] and perhaps the majority of studies are at low risk of bias, however the previous studies [43,44] were conducted in highly regulated fields and it would be inappropriate to generalise to the area of chronic wounds trials. Even under the most optimistic scenario (that the unclear risk trials reflect poor reporting rather poor trial methodology) there remains a substantial risk of bias in chronic wounds trials due to almost a third of trials not blinding outcome assessors.

The methodological quality of commercially funded trials and non-commercially funded trials was not significantly different across all the included measures of methodological quality. Based on current evidence it is not possible to draw the conclusion regarding the influence of funding source on the methodological quality of chronic wounds trials, although we acknowledge the limited power in these analyses that in turn also limits the conclusions that can be drawn. Furthermore, the overall standard of trial quality was very poor and wound trialists share a culture that international trial design, conduct and reporting standards do not seem to have penetrated. Almost a quarter of the sample had an unclear funding source; more transparency in declaring support for research will allow more studies to be included in future analysis of this type.

### Comparison with other studies

Our results are very similar to those obtained in other methodological reviews that used samples of trials from across medicine (for example, Hopewell *et al*. [16]). Chronic wounds trials are therefore not considerably methodologically weaker than trials conducted in some other areas of medicine. The cohort assessed in Hopewell *et al.*[16], however, was much older and dated back to 2000 and this study includes trials up to 2011, thus limiting comparability, particularly as the impact of CONSORT may mean that trials are now of higher methodological quality than observed by Hopewell *et al*. [16]. It should also be noted that the methodological quality of reports assessed by Hopewell *et al*. [16] was poor and the fact that chronic wounds trials may be of comparable quality is no reason for complacency or celebration.

### Limitations

The initial selection of studies was carried out by a single reviewer and lack of translation services means that only English language papers were included. As a result our sample may not be fully representative of all the chronic wounds trials published during the period 2004 to 2011. While it is difficult to predict how inclusion of non-English language studies would have impacted the findings, evidence from other fields suggest that methodological quality is similar or slightly higher in English language publications, compared with those published in other language [45-48]. On this basis, our results would represent a conservative assessment of methodological quality of chronic wounds trials.

A further limitation of this study is that it was based only on information contained within trial reports and does not necessarily mean poor trial conduct. Studies [43,44] have compared the content of published reports with their protocol and found that the methodological techniques in the actual trial were of better quality than reported in the final publication. However, these focused on specific areas of medicine, and as such, the results may not be generalizable to a chronic wounds context. Assessment of review protocols and contacting trialists for more information may have revealed that the trials were of better quality than reported in the final publication. This was not done in this study.

## Conclusions

This methodological review of recent chronic wounds trials presents a profile of the recent trial literature in this area. It identifies that chronic wounds trials tend to suffer from a number of methodological deficiencies, with trials tending to be small and underpowered, with short follow-up periods, widespread use of surrogate healing measures and frequently no primary outcome specified. Further, on the basis of Cochrane review guidelines, a significant proportion of trials would be considered to be at either unclear or at high risk of bias.

The fact that such a large proportion of the trial evidence base is methodologically weak inevitably has a huge impact on the potential for current chronic wounds trials to inform practice. Given the considerable expense of conducting RCTs and the ethical implications of recruiting participants to methodologically weak studies, much greater efforts must be made by researchers to ensure that best practice is followed wherever possible. In particular, much greater efforts must be made to blind outcome assessors [49]. While there are occasions where blinding of assessors is not possible, it is important to emphasise that blinding is possible in wounds trials as has been demonstrated in a number of studies, often by using photographic assessment by masked adjudicators [33,50,51].

While funding source was not associated with greater risk of bias, the unfortunate reality is that, with few exceptions, chronic wounds trials are unacceptably poor across a whole range of measures of methodological quality. In the light of these findings the publication of methodological guidelines for comparative effectiveness research in chronic wounds by the Centre for Medical Technology Policy represents a positive step in improving the methodological quality of chronic wounds trials [52]. Moving forward, it is incumbent upon all stakeholders in chronic wounds research, irrespective of funding source, to insist on the improvements and recommendations outlined in these guidelines. If improvements are not made, there will continue to be a deficit in high quality evidence available to inform practitioners’ decisions regarding the best treatment options for their patients.

## Abbreviations

CI: Confidence interval; IQR: Interquartile range; OR: Odds ratio; PO: Primary outcome; RCT: Randomised controlled trial.

## Competing interests

The authors declare that they have no competing interests.

## Authors’ contributions

RH, JD, RA, SBS, RF, JH, KL, MM, SOM, NS and NS were involved in the conception and design of the study. RF carried out the necessary searches for the project. RJA, EB, JD, RLA, SBS, RF, JH, KL, SOM, NS and NC completed the data collection and extraction process coordinated by RH. RH and RJA with guidance from MB conducted data analysis for the paper. RH drafted the first version of the manuscript. All authors have commented extensively and approved the final version.

## Supplementary Material

Additional file 1 Online Supplementary Content. Click here for file

## References

[B1] Mustoe T Understanding chronic wounds: a unifying hypothesis on their pathogenesis and implications for therapy Am J Surg 2004 187 65S 70S 10.1016/S0002-9610(03)00306-4 15147994

[B2] Watson J Kang’ombe A Soares M Chuang L Worthy G Bland M Iglesias C Cullum N Torgerson D Nelson E VenUS III: a randomised controlled trial of therapeutic ultrasound in the management of venous leg ulcers Health Technol Assess 2011 15 1 176 10.3310/hta15130 21375959

[B3] Walker A Nixon J Improving the quality of reporting in randomised controlled trials J Wound Care 2004 13 103 106 15045804 10.12968/jowc.2004.13.3.26593

[B4] Werdin F Tennenhaus M Schaller H Rennekampff H Evidence-based management strategies for treatment of chronic wounds Eplasty 2009 9 e19 19578487 PMC2691645

[B5] Gottrup F Apelqvist J Price P Outcomes in controlled and comparative studies on non-healing wounds: recommendations to improve the quality of evidence in wound management J Wound Care 2010 19 237 268 20551864 10.12968/jowc.2010.19.6.48471

[B6] Kjaergard L Villumsen J Gluud C Reported methodological quality and discrepancies between large and small randomized trials in meta-analyses Ann Intern Med 2001 135 982 989 10.7326/0003-4819-135-11-200112040-00010 11730399

[B7] Jüni P Altman D Egger M Systematic reviews in health care: assessing the quality of controlled clinical trials BMJ 2001 323 42 46 10.1136/bmj.323.7303.42 11440947 PMC1120670

[B8] Egger M Juni P Bartlett C Holenstein F Sterne J How important are comprehensive literature searches and the assessment of trial quality in systematic reviews: empirical study Health Technol Assess 2003 7 1 12583822

[B9] Woods L Egger M Gluud L Schulz K Jüni P Altman D Gluud C Martin R Wood A Sterne J Empirical evidence of bias in treatment effect estimates in controlled trials with different interventions and outcomes: meta-epidemiological study BMJ 2008 336 601 605 10.1136/bmj.39465.451748.AD 18316340 PMC2267990

[B10] Nüesch E Reichenbach S Trelle S Rutjes S Liewald K Sterchi R Altman D Jüni P The importance of allocation concealment and patient blinding in osteoarthritis trials: a meta-epidemiologic study Arthritis Rheumatism 2009 61 1633 1641 10.1002/art.24894 19950329

[B11] Hróbjartsson A Thomsen A Emanuelsson F Tendal B Hilden J Boutron I Ravaud P Brorson S Observer bias in randomised clinical trials with binary outcomes: systematic review of trials with both blinded and non-blinded outcome assessors BMJ 2012 344 e1119 10.1136/bmj.e1119 22371859

[B12] Savovic’ J Jones H Altman D Harris RJ Jüni P Pildal J Als-Nielsen B Balk E Gluud C Gluud L Ioannidis J Schulz K Beynon R Welton N Wood L Moher D Deeks J Sterne J Influence of reported study design characteristics on intervention effect estimates from randomized controlled trials Ann Intern Med 2012 157 429 438 10.7326/0003-4819-157-6-201209180-00537 22945832

[B13] Kirkham J Dwan K Altman D Gamble C Dodd S Smyth R Williamson P The impact of outcome reporting bias in randomised controlled trials on a cohort of systematic reviews BMJ 2010 340 c365 10.1136/bmj.c365 20156912

[B14] Singh J Murphy S Bhandari M Trial sample size, but not trial quality, is associated with positive study outcome J Clin Epidemiol 2010 63 154 162 10.1016/j.jclinepi.2009.05.007 19716266 PMC2818000

[B15] Chan A Altman D Epidemiology and reporting of randomised trials published in PubMed journals Lancet 2005 365 1159 1162 10.1016/S0140-6736(05)71879-1 15794971

[B16] Hopewell S Dutton S Yu L Chan A Altman D The quality of reports of randomised trials in 2000 and 2006: comparative study of articles indexed in PubMed BMJ 2010 340 c723 10.1136/bmj.c723 20332510 PMC2844941

[B17] Hui D Arthur J Dalal S Bruera E Quality of the supportive and palliative oncology literature: a focused analysis on randomized controlled trials Support Care Cancer 2012 20 1779 1785 10.1007/s00520-011-1275-9 21935717

[B18] Shuai P Zhou X Lao L Li X Issues of design and statistical analysis in controlled clinical acupuncture trials: an analysis of English-language reports from Western journals Stat Med 2010 31 606 618 21341295 10.1002/sim.4034 PMC3631592

[B19] Strippoli G Craig J Schena F The number, quality and coverage of randomized controlled trials in nephrology J Am Soc Nephrol 2004 15 411 419 10.1097/01.ASN.0000100125.21491.46 14747388

[B20] Danilla S Wasiak J Searle S Arrigada C Pedreros C Cleland H Spinks A Methodological quality of randomised controlled trials in burns care A Syst Rev Burns 2009 35 956 961 10.1016/j.burns.2009.04.031 19545949

[B21] Yaphe J Edman R Knishkowy B Herman J The association between funding by commercial interests and study outcome in randomized controlled drug trials Fam Pract 2001 18 565 568 10.1093/fampra/18.6.565 11739337

[B22] Lexchin J Bero L Djulbegovic B Clark O Pharmaceutical industry sponsorship and research outcome and quality: systematic review BMJ 2003 326 1167 1170 10.1136/bmj.326.7400.1167 12775614 PMC156458

[B23] Perlis R Perlis C Wu Y Hwang C Joseph M Nierenberg A Industry sponsorship and financial conflict of interest in the reporting of clinical trials in psychiatry Am J Psychiatry 2005 162 1957 1960 10.1176/appi.ajp.162.10.1957 16199844

[B24] Lundh A Sismondo S Lexchin J Busuioc O Bero L Industry sponsorship and research outcome Cochrane Database of Syst Rev 2012 doi:10.1002/14651858.MR000033.pub2 10.1002/14651858.MR000033.pub2 23235689

[B25] Ioannidis J Effect of the statistical significance of results on the time to completion and publication of randomized efficacy trials JAMA 1998 279 281 286 10.1001/jama.279.4.281 9450711

[B26] Mandelkern M Manufacturer support and outcome J Clin Psychiatry 1999 60 122 123 10084640 10.4088/jcp.v60n0210a

[B27] Djulbegovic B Lacevic M Cantor A Fields K Bennett C Adams J Kuderer N Lyman G The uncertainty principle and industry-sponsored research Lancet 2000 356 635 638 10.1016/S0140-6736(00)02605-2 10968436

[B28] Cho M Bero L The quality of drug studies published in symposium proceedings Ann Intern Med 1996 124 485 489 10.7326/0003-4819-124-5-199603010-00004 8602706

[B29] Kjaergard L Nikolova D Gluud C Randomized clinical trials in Hepatology: predictors of quality Hepatology 1999 30 1134 1138 10.1002/hep.510300510 10534332

[B30] Jones R Younie S Macallister A Thornton J A comparison of the scientific quality of publicly and privately funded randomized controlled drug trials J Eval Clin Pract 2010 16 1322 1325 10.1111/j.1365-2753.2009.01335.x 20738476

[B31] Higgins J Altman D Gotzsche P Juni P Moher D Oxman A Savovic J Schulz K Weeks L Sterne J Cochrane Bias Methods Group The Cochrane Collaboration’s tool for assessing risk of bias in randomised trials BMJ 2011 343 d5928 10.1136/bmj.d5928 22008217 PMC3196245

[B32] Higgins J Green S Cochrane Handbook for Systematic Reviews of Interventions Version 5.1.0 [http://www.cochrane-handbook.org]

[B33] Iglesias C Nelson E Cullum N Torgerson D on behalf of the VenUS Team VenUS I: a randomised controlled trial of two types of bandage for treating venous leg ulcers Health Technol Assess 2004 8 1e120 10.3310/hta8290 15248939

[B34] Halpern S Karlawish J Berlin J The continuing unethical conduct of underpowered clinical trials JAMA 2002 288 358 362 10.1001/jama.288.3.358 12117401

[B35] Bacchetti P Current sample size conventions: flaws, harms, and alternatives BMC Med 2010 8 17 10.1186/1741-7015-8-17 20307281 PMC2856520

[B36] Norman G Monteiro S Salama S Sample size calculations: should the emperor’s clothes be off the peg or made to measure? BMJ 2012 345 e5278 10.1136/bmj.e5278 22918496

[B37] Lockyer S An investigation into the reporting and interpretation of randomised controlled trials in wound care where the primary outcomes are statistically non-significant or unclear 2012 MSc. thesis: University of York, Department of Health Sciences

[B38] Cardinal M Eisenbud D Phillips T Harding K Early healing rates and wound area measurements are reliable predictors of later complete wound closure Wound Repair Regen 2008 16 19 22 10.1111/j.1524-475X.2007.00328.x 18211575

[B39] Edsberg L Wyffels J Daniel H Longitudinal Study of Stage III and Stage IV Pressure Ulcer Area and Perimeter as Healing Parameters to Predict Wound Closure Ostomy Wound Manag 2011 57 50 62

[B40] O’Meara S Bland M Dumville J Cullum N A systematic review of the performance of instruments designed to measure the dimensions of pressure ulcers Wound Repair Regen 2012 20 263 276 10.1111/j.1524-475X.2012.00783.x 22564222

[B41] Gelfand J Hoffstad O Margolis D Surrogate endpoints for the treatment of venous leg ulcers J Investig Dermatol 2002 119 1420 1425 10.1046/j.1523-1747.2002.19629.x 12485449

[B42] Berger V Does the Prentice criteria validate surrogate endpoints? Stat Med 2004 23 1571 1578 10.1002/sim.1780 15122737

[B43] Devereaux P Choi P El-Dika S Bhandari M Montori V Schunemann H Garg A Busse J Heels-Ansdell D Ghali W Manns B Guyatt G An observational study found that authors of randomized controlled trials frequently use concealment of randomization and blinding, despite the failure to report these methods J Clin Epidemiol 2004 57 1232 1236 10.1016/j.jclinepi.2004.03.017 15617948

[B44] Soares H Daniels S Kumar A Clarke M Scott C Swann S Djulbegovic B Radiation Therapy Oncology Group Bad reporting does not mean bad methods for randomised trials: observational study of randomised controlled trials performed by the Radiation Therapy Oncology Group BMJ 2004 328 22 24 10.1136/bmj.328.7430.22 14703540 PMC313900

[B45] Egger M Zellweger-Zahner T Schneider M Junker C Lengeler C Antes G Language bias in randomised controlled trials published in English and German Lancet 1997 350 326e9 9251637 10.1016/S0140-6736(97)02419-7

[B46] Moher D Fortin P Jadad A Juni P Klassen T Le Lorier J Liberati A Linde K Penna A Completeness of reporting of trials published in languages other than English: implications for conduct and reporting of systematic reviews Lancet 1996 347 363e6 8598702 10.1016/s0140-6736(96)90538-3

[B47] Juni P Holenstein F Sterne J Bartlett C Egger M Direction and impact of language bias in meta-analyses of controlled trials: empirical study Int J Epidemiol 2002 31 115e23 11914306 10.1093/ije/31.1.115

[B48] Shiwa S Moseley A Maher C Pena Costa L Language of publication has a small influence on the quality of reports of controlled trials of physiotherapy interventions J Clin Epidemiol 2013 66 78 84 10.1016/j.jclinepi.2012.08.004 23177897

[B49] Eskes AM Brölmann FE Sumpio BE Mayer D Moore Z Agren MS Hermans M Cutting K Legemate DA Ubbink DT Vermeulen H Fundamentals of randomized clinical trials in wound care: design and conduct Wound Repair Regen 2012 20 449 455 22642397 10.1111/j.1524-475X.2012.00799.x

[B50] Hollisaz M Khedmat H Yari F A randomized clinical trial comparing hydrocolloid, phenytoin and simple dressings for the treatment of pressure ulcers BMC Dermatol 2004 4 18 10.1186/1471-5945-4-18 15601464 PMC545970

[B51] Dumville J Worthy G Soares M Bland M Cullum N Dowson C Iglesias C McCaughan D Mitchell JL Nelson EA Torgerson DJ VenUS II team VenUS II: a randomised controlled trial of larval therapy in the management of leg ulcers Health Technol Assess 2009 13 1 182 19925723 10.3310/hta13550

[B52] Sonnad S Goldsack J Mohr P Whicher D Methodological Recommendations for Comparative Effectiveness Research on the Treatment of Chronic Wounds [http://www.cmtpnet.org/wp-content/uploads/downloads/2012/10/Wound-Care-2012.pdf] 10.12968/jowc.2013.22.9.470 24005781

