# Supplementary material for: Funding source and the quality of reports of chronic wounds trials: 2004 to 2011
Source: Trials. 2014 Jan 14;15:19. doi: 10.1186/1745-6215-15-19 (PMC3896781; doi:10.1186/1745-6215-15-19)
Supplement: Additional file 1 — Online Supplementary Content. [file 1745-6215-15-19-S1.docx]

## Additional File 1: Decision rules used for data classification

## Funding Type

The decision regarding funding source will be based on published disclosures of full or partial funding.

When classifying funding source in the absence of a funding disclosure in study reports, we will consider any of the following as constituting funding by a commercial organization (that we will take a conservative approach):

- declarations of consulting
- speaking fees
- honoraria
- stock ownership
- commercial funding of study product in absence of other commercial funding for the study

Where a paper explicitly states the study is independent, non-commercial funding will be recorded, even if the funding source is not provided (as long as there is no evidence in the paper that authors were employed by a commercial organization). In the absence of a funding disclosure, employment of an author by commercial enterprise will be considered commercial funding even in the presence of any other funding statements. Where a conflict of interest statement is recorded as none, non-commercial will be recorded even if the funding source is not reported.

Where no funding information is found in the paper, the reviewers will check the *International Standard Randomized Controlled Trial Number* database for a record of the study. Where the Source of Funding’ field contains relevant information this will be extracted. If funding details are not in the paper and not found on this database then ‘not reported’ will be recorded.

In situations where the reviewer is unsure how to class a funding body that had been given in a report, a Google search will be conducted to locate further information to help establish whether the body was commercial or non-commercial. If there is no further information Unclear will be reported. If information is found the two reviewers will discuss the nature of the funder and classify. Where they are unable to make a decision regarding a funder’s status details will be discussed with a third reviewer. A central log of funders will be kept for reference so that all reviewers can ensure consistency.

## Journal Type

Journals will be categorised as General Medicine, Wounds Journal or Other Speciality Journal, using data from the National Library of Medicine (NLM). Journals will classified according to the following rules:

General Medicine – if the journal has MeSH label of “Medicine” (N.B. it may not have any other MeSH classification attached if it does it will be classified as a Wounds Journal or Other speciality according to the rules below).

Wounds Journal – if the journal has a MeSH label of “Wounds and Injuries” or “Wound Healing” (N.B. it may also have other MeSH headings).

Other Speciality – if the journal does not fit in the other two categories i.e. it does not have a MeSH label of “Medicine” only, or MeSH label of “Wounds and Injuries” or “Wound Healing”.

Journal type will be assessed after extraction has been completed from which a list of journals in which included studies were published will be complied. The NLM will then be used to ascertain the associated journal type on a single yet to be determined date which will be recorded.

##

## Impact Factor

The impact factor will be recorded, using data from Journal Citation Reports (Web of Knowledge). Where no impact factor is available this will be recorded as NA. Impact factor will assessed after extraction has been completed from which a list of journals in which included studies were published will be complied. The Journal of Citation reports will then be used to ascertain the associated impact factor on a single yet to be determined date which will be recorded.
